# News and Miscellany

**Published:** 1903-07

**Authors:** 


### News and Miscellany.

---

CLUB FOOT OPERATION.—We saw our esteemed contemporary of *The Texas Medical News,* Dr. M. M. Smith, of Austin, operate for *talipes equino varus* at Seton Infirmary on the 26th ult. before the members of the Austin District Medical Society. It was a child of two and a half years, and was a most gratifying success. Dr. Smith and Dr. Knox of Houston operated—one on each foot—on a case of double club-foot at Victoria during the meeting of the South Texas Medical Society. The Texas doctors have "caught on" and the operation is now done by Texas surgeons in several parts of the State as well as Lorenz does it.

---

"DO HOGS PAY?" asks a farmers' journal. Lamphear, the inimitable, who spells "kissed" *"kist,"* says that in his experience they do not; that they take a medical journal about five years without paying for it, and then return a copy to the publisher marked "refused." We have had some experience of that kind. No, hogs don't pay.

---

WILL REPAIR YOUR ELECTRICAL MACHINES.—Static and all electrical medical apparatus put in running order. I am also agent for electrical and X-ray apparatus. Oliver Brush, 710 Colorado Street, Austin, Texas.

---

EXAMINATION of urine, sputum, blood, pathological specimens, etc., made at reasonable rates by New Orleans Clinical Laboratory, 124 Barrone Street, New Orleans. O. L. Pothier, M. D., Charity Hospital. I. I. Lemann, M. D., Secretary. J. B. Guthrie, M. D.

Dr. M. K. Lott, of Cameron, widely known in connection with his painless treatment of the drug habit, was in a wreck on the T. P. R. R., near Jefferson, Texas, June 4th, and sustained, we learn, serious injuries.

---

For Sale.—Residence worth $1500, $600 stock of drugs, $2500 practice, all for $1500. A bargain for the right man. Situated in county seat west of Austin. Write at once to "B," care Texas Medical Journal, Austin, Texas.

---

Death of Dr. Orpheus Edwards.—This distinguished physician died at his home at College Hill, Cincinnati, June 19th (ult.), aged 77 years. Dr. Edwards had been for many years superintendent of the noted sanitarium at College Hill.

---

Dr. E. L. Thompson, of Dallas, Texas, one of the most widely known physicians of Texas, died suddenly at Mineral Wells June 12th. His death was a great surprise to his friends, as he was the very picture of good health. He was a graduate of the New Orleans School of Medicine in the class of 1860-61. Dr. Thompson had formerly lived at Chappell Hill, Texas, where he and the ever lamented Swearingen were partners.

---

Ex-State Health Officer W. F. Blunt died at his home in Lockhart, Texas, June 25th. Dr. Blunt was a native of Bellfield, Va., and was in his sixty-sixth year. The Public Health Department of Texas was closed and many friends from the various sections of the State went down to attend the funeral.

---

The Strange Case of Dr. Bruno, a Novel, by F. E. Daniel. I have reconsidered my determination, as expressed in my May issue, to publish my book serially in the "Red Back." It will be issued in book form October 1st in same handsome binding as "Recollections of a Rebel Surgeon," scarlet cloth and gilt. Price, delivered, $1. To be sold by subscription only. Send your $1 now and secure a copy. Only 500 will be issued.

---

Chacun a Son Gout.—Dr. W. T. Bull called in consultation a negro physician named Wheatland in the case of a millionaire named McKay. Wheatland supplanted him in the bosom of some other millionaire patrons cuckoo fashion, and, according to the press dispatches, he is now the latest fad with the plutocrats and is the social lion at Newport. Look to your laurels, Strenuous and Booker.

---

The Alvarenga Prize was awarded by the College of Physicians of Philadelphia to our own Dr. W. S. Carter, Professor of Physiology in the Medical Department of the University of Texas, Galveston. Texas is proud of Dr. Carter. This triumph of one of her young sons reflects great credit upon the State. This prize was

### DALLAS MEDICAL COLLEGE.

**Fourth Session Opens October 1, 1903, and Continues Six Months.**

**The Largest Medical College in the Great Southwest. New Buildings and Equipment. Ample Hospital Facilities. A Large Faculty of Salaried Teachers.**

While the tuition fees are slightly in advance of the other southern medical schools the advantages are greater.

**Write for Particulars.**

**HUGH L. McNEW, M. D., Dean.**

**DALLAS, TEXAS.**

awarded a few years ago to another Texas physician, Dr. R. H. L. Bibb, now Chief Surgeon of the Mexican National Railroad at Saltillo. Texas heads the procession.

---

Boom! Boom!—Organization is booming in Texas. Travis county organized on the 17th inst. with nearly every physician in the county in attendance. Dr. B. M. Worsham, Superintendent of the State Lunatic Asylum, was elected president. Williamson county organized recently with a membership of fifty-nine. Dr. W. T. Jones, Georgetown, president. Want of space prevents more extended notice, as we are just going to press. Keep it up, boys, and at Austin next April let us have a thousand or more in attendance.

